# Hepatitis C in Hemodialysis Units: diagnosis and therapeutic approach

**DOI:** 10.1590/2175-8239-JBN-2018-0177

**Published:** 2019-02-18

**Authors:** Natasha Silva Constancio, Maria Lucia Gomes Ferraz, Carmen Tzanno Branco Martins, Angiolina Campos Kraychete, Paulo Lisboa Bitencourt, Marcelo Mazza do Nascimento

**Affiliations:** 1 Associação Renal Vida Rio do Sul, Rio do Sul, SC, Brasil.; 2 Universidade Federal de São Paulo, São Paulo, SP, Brasil.; 3 Universidade Federal do Paraná, Curitiba, PR, Brasil.; 4 Sociedade Brasileira de Hepatologia, São Paulo, SP, Brasil.; 5 Sociedade Brasileira de Nefrologia, São Paulo, SP, Brasil.

**Keywords:** Hepatitis C, Renal Dialysis, Renal Insufficiency, Chronic, Hepatitis Viruses, Antiviral Agents, Hepatite C, Diálise Renal, Insuficiência Renal Crônica, Vírus de Hepatite, Antivirais

## Abstract

According to data from the last census of the Brazilian Society of Nephrology (SBN), the prevalence of hepatitis C virus (HCV) in Brazilian hemodialysis units (HU) is 3.3%, about three times higher than what is reported for the Brazilian general population. Often, professionals working in HU are faced with clinical situations that require rapid HCV diagnosis in order to avoid horizontal transmission within the units. On the other hand, thanks to the development of new antiviral drugs, the cure of patients with HCV, both in the general population and in patients with chronic kidney disease and the disease eradication, appear to be very feasible objectives to be achieved in the near future . In this scenario, SBN and the Brazilian Society of Hepatology present in this review article a proposal to approach HCV within HUs.

## Resumo

De acordo com os dados do último censo da Sociedade Brasileira de Nefrologia (SBN), a prevalência de portadores do vírus da hepatite C (HCV) nas unidades de hemodiálise (UH) no Brasil é de 3,3%, cerca de três vezes maior do que é observado na população geral brasileira. Muitas vezes, os profissionais que trabalham nas UH deparam-se com situações clínicas que demandam rápido diagnóstico do HCV, a fim de evitar uma transmissão horizontal dentro das unidades. Por outro lado, a cura dos pacientes portadores do HCV, tanto na população geral como na portadora de doença renal crônica e a erradicação da doença, em virtude do desenvolvimento de novas drogas antivirais, parecem ser objetivos bastante factíveis, a ser alcançados em futuro próximo. Nesse cenário, a SBN e a Sociedade Brasileira de Hepatologia apresentam neste artigo de revisão uma proposta de abordagem do HCV dentro das UH.

## Introduction

Since its identification in 1989 by Choo et al.,^1^ the hepatitis C virus (HCV) has been causing concern in the scientific community because of the development of both acute and chronic liver disease, significantly increasing the risk of cirrhosis and hepatocellular carcinoma. Epidemiological data indicate that about 170 million people have chronic HCV infection.^2^^-^ 4 Regardless of country of origin, hepatitis C prevalence is higher in hemodialysis (HD) patients,^5^^-^ 7 and its prevalence in different geographic regions vary widely, from 4% in England to more than 70% in regions such as Kuwait and Cuba.^7^ According to data from the last SBN census of 2017, the prevalence in Brazil is 3.3%, about three times higher than that reported in the Brazilian general population,^8^^,^ 9 although a study that specifically evaluated the C virus epidemiology and genotyping in dialysis patients in Brazil has shown an even higher prevalence of 8.4%.^10^ Although high, these percentages are well below the 15.4% prevalence detected in this population 16 years ago.^11^ Still in agreement with US data available on the Centers for Disease Control (CDC) website, more than half of the hepatitis C outbreaks from 2008 to 2015 occurred in HU settings,^12^ noting that the risk of HCV infection increases as patient stay more time in HD.^6^

Unlike hepatitis B, the development of a hepatitis C vaccine has not yet been possible.^4^ The challenge is even greater for many nephrologists because of the difficulty in diagnosing chronic C virus infection in dialysis patients because of the lower sensitivity of the diagnostic tests in this population.^7^ Significant progress has been made in the last decade, culminating in a marked improvement in the treatment of HCV infection. Levels above 90% of sustained virological response (SVR) have been reached, including in the CKD population.^13^^,^ 14 Today there is a real promise of eliminating hepatitis C in the next 15 to 20 years, but although we are experiencing a new era in relation to this disease, better knowledge about the number and characteristics of infected patients is needed to plan strategies for its eradication.^3^

Therefore, the Brazilian Society of Nephrology and the Brazilian Society of Hepatology propose in this article a guideline for HCV screening, the adoption of preventive measures within Dialysis Units (HU) and the therapeutic approach of dialysis patients in our country.

## Discussion

### Diagnostic tests

#### Serological tests

**Figure 1:**
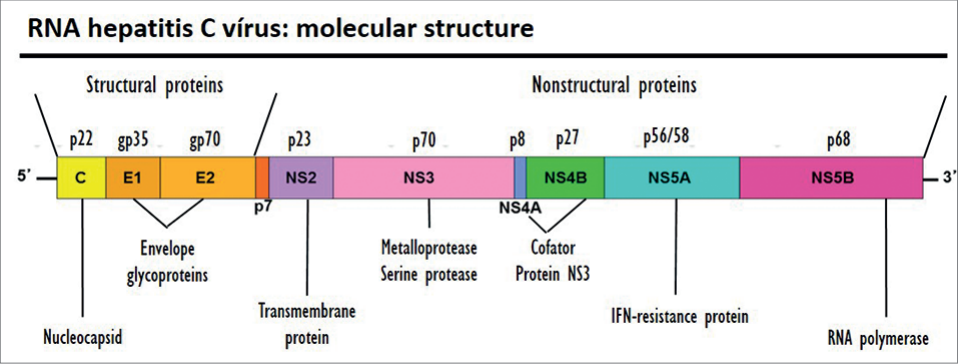
Hepatitis C virus genome and structure.

HCV is an RNA virus that has 6 genotypes and multiple subtypes (Figure 1). The prevalence of each genotype varies according to geographic region, with genotypes 1a and 1b being the most prevalent in the United States and Europe, followed by genotypes 2 and 3; whereas in Egypt the genotype 4 is the preponderant one; in South Africa, genotype 5; and the genotype 6 in Southeast Asia.^15^ In Brazil, genotypes 1, 2 and 3 predominate.^16^

After cloning the HCV genome, scientists managed to determine the various viral proteins and antigenic regions and epitopes. Recombinant proteins and synthetic peptides, containing these dominant epitopes, were used in the development of immunoassays, which enable the detection of the anti-HCV IgG immunoglobulin. The currently used 3rd generation immunoenzymatic assays (IEA) determine specificity of up to 99% and reduction in the immunological window period by approximately 5 weeks compared to 1st generation assays.^17^

RIBA (recombinant immunoblot assay) assays have emerged as more specific alternatives for anti-HCV detection than immunoassays, based on recombinant peptides from specific antigenic regions, but they are not used in routine clinical practice, since, like the ELISA tests, they do not enable the differentiation between active and resolved infection, and its cost is high.

#### RNA analysis tests

Even if 3rd generation tests are performed, immunoassays may present false negative results in immunocompromised and in HD patients.^17^ HCV nucleic acid (HCV-RNA) detection remains the gold standard in the diagnosis of active infection. Despite the excellent sensitivity and specificity, it is a more expensive and not always available test.

Detection of HCV-RNA by nucleic acid (NAT) assays, by the polymerase chain reaction (PCR) or transcription-mediated amplification (TMA) method, can rapidly detect HCV infection, within approximately 1 week post-exposure by comparison to 10 weeks of the 3rd generation IEA. All NAT-based tests approved for clinical use have specificity above 99% for the 6 genotypes and viral load detection levels from 12 IU/mL and 10 IU/mL for PCR and TMA, respectively. Studies to confirm the phenomenon of intermittent viremia occurring in hemodialysis patients have demonstrated the importance of diagnostic evaluation in more than one determination using molecular methods in patients initially considered non-viraemic.^18^ Serum HCV-RNA can be significantly reduced during hemodialysis sessions; therefore, blood sample collection should always be performed before the dialysis session.^19^^,^ 20

## HCV tracking in the dialysis room

**Figure 2:**
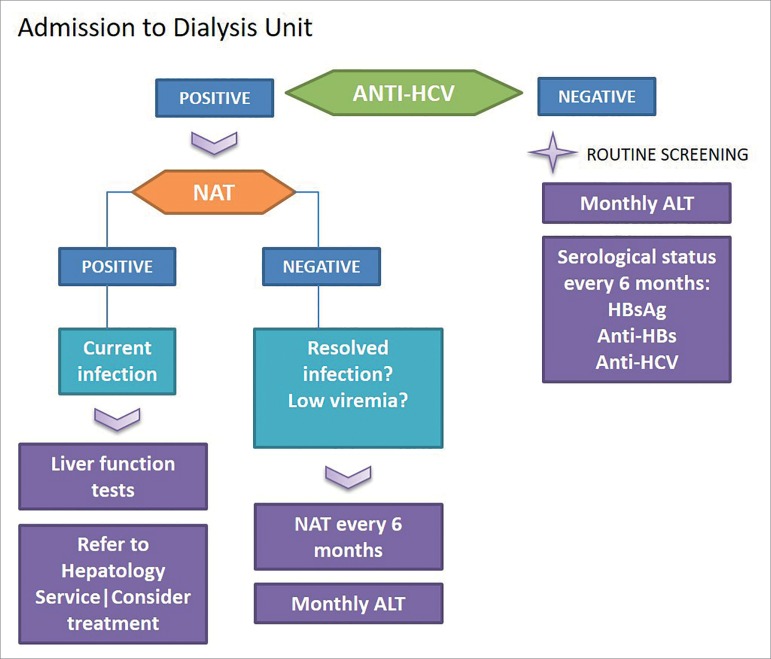
Initial HCV serological assessment flowchart after admission to the Dialysis Unit.

**Figure 3:**
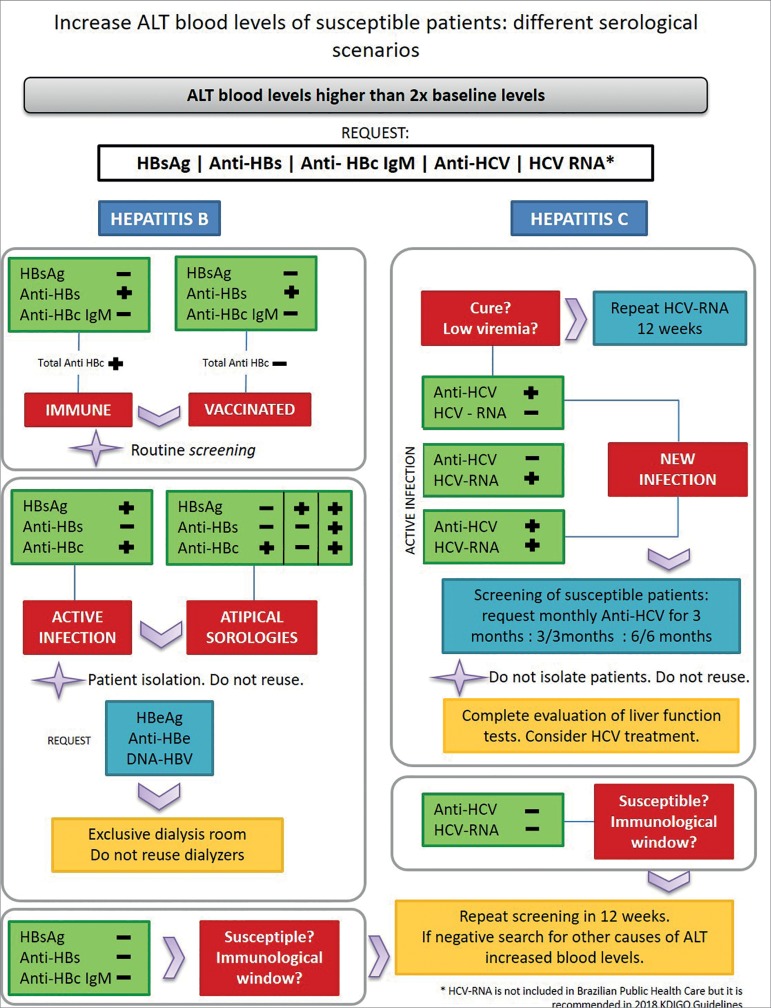
Follow-up serum flowchart in the Hemodialysis Unit.

Screening for hepatitis C should be performed in all patients who initiate the dialysis program or are transferred from other centers, initially with immunoassay, and if positive, confirmed by NAT. However, in countries with a high hepatitis C prevalence, methods for the detection of HCV-RNA may be considered as the initial examination.^21^^,^ 22 Figures 2 and 3 depict suggestions for conducting the initial assessment and serological follow-up of patients in HD program.

Hepatitis C diagnosis in patients with chronic kidney disease (CKD) is difficult due to some reasons, such as: presence of nonspecific clinical signs and symptoms, being often asymptomatic; normal or discrete (often fluctuating) levels of the alanine aminotransferase (ALT) enzyme in almost half of the patients with HCV infection; presence of possibly false-negative serology, in addition to the low viremia seen in these patients.^23^ ALT levels should be checked on admission to the dialysis unit and then monthly. Recently infected patients may have elevated ALT levels prior to seroconversion, which warrants monitoring levels for early detection of new infections.^19^ Patients with unwarranted alteration of ALT, even if discrete elevations, should be investigated for hepatitis B and C. The validity or usefulness of monthly ALT dosing in patients with chronic hepatitis C infection resolved is unknown and there are no specific recommendations for this subgroup.^21^^,^ 22

It has been shown that dialytic patients may have lower aminotransferase levels than individuals with normal renal function, but the reasons for this fact remain uncertain. The main hypotheses for this reduction is the hemodilution (which would alter the dosage of liver enzymes) or the reduced levels of pyridoxine or elevated homocysteine.^24^^,^ 25 On the other hand, HCV-infected HD patients have higher aminotransferase levels than their uninfected counterparts. A study conducted to evaluate the predictive value of ALT dosing for HCV infection in HD patients showed inadequate accuracy of the test, although widely used, with sensitivity and specificity for new infections of 83% and 90%, respectively. Changes in the cut-off value may help to improve accuracy, but the adequate value has not yet been defined, ranging from 45% to 70% of the upper limit of normality.^20^^,^ 26 In order to improve the diagnostic performance of the enzyme, studies suggest that the value of ALT found in patients on hemodialysis be increased by 50% of its baseline value.^27^

Some factors may reduce the production of antibodies against HCV surface antigens, such as the immunosuppressive effect of chronic uremia, high concentrations of proinflammatory cytokines and diabetes, which may explain the false negative serological tests, even when this occurrence is rare.^28^ Another point of concern is the phenomenon of intermittent viremia, which is not only epidemiological, but also an inconvenience in the control of nosocomial transmission, since the results can be misinterpreted, classifying a patient with active infection as non-virememic.^18^^,^ 20 ^,^ 29 ^,^ 30 The use of molecular tests with low detection levels should always be recommended.

Currently, the Ministry of Health recommends that all patients who initiate HD should be submitted routinely to monthly ALT analysis and serological profile analysis by performing anti-HCV upon dialysis onset, and every six months thereafter. In the year 2018, Kidney Disease Improving Global Outcomes (KDIGO) published the recommendations regarding the management of HCV in patients with CKD on dialysis: all patients not infected by HCV with negative anti-HCV tests should be monitored every 6 months in relation to their serological profile; while HCV-RNA negative and HCV-positive patients (infection resolved but at risk of reinfection) should be monitored by NAT every six months or whenever there is an ALT elevation.^19^ The same guideline indicates that positive anti-HCV patients should be submitted to the NAT every six months, to look for viremia.

Although some studies have shown benefits in performing molecular tests for the early detection of acute C virus infection in a dialysis unit, this recommendation becomes difficult to apply in our country, taking into account its high cost to detect virus RNA. Data available since 1999 shows a prevalence of less than 2% of HCV-RNA positive patients with anti-HCV negative testing; with more recent studies showing even lower false-negative rates, ranging from 0.1 to 0.86%, confirming that immunoassay is a reliable method to be used as screening.^7^^,^ 23 The NAT test is always indicated in cases of a positive anti-HCV result. It is recommended that patients with anti-HCV positive and HCV-negative RNA necessarily need screening (or follow-up in the HU) using NAT.^31^

Acute HCV infection should be reported to the local Epidemiological Surveillance team. Acute cases are those with negative anti-HCV or HCV-RNA serology and subsequent positive serological examination. A mild elevation of ALT is often the first sign of an acute infection, and should be appreciated. A new case in a dialysis unit should immediately trigger actions to identify additional cases, with serological reassessment of all uninfected. The screening frequency on this unit should also be changed for a set time. One suggestion is to reduce the anti-HCV testing frequency in all susceptible to monthly for 3 months or NAT in the patients on the same dialysis session and who initially showed a 50% transaminase elevation in relation to their baseline values. If there is no seroconversion, retest in 3 months. In the absence of any new cases identified, the HD routine of six-month serology can be returned.^18^^,^ 23

## Preventive measures

HCV is transmitted parenterally through percutaneous exposure to contaminated blood. Rigorous screening policies in blood donors and widespread use of erythropoiesis have reduced the incidence of blood transfusion, and today the main route of transmission is nosocomial.^22^ According to data from the Centers of Disease Control, more than 50% of outbreaks of hepatitis C in the United States between 2008 and 2015 were related to HU.^12^

Studies published in the early 2000s have shown significant declines in the incidence of HCV infection horizontal transmission through only the adoption of universal measures in many European clinics that did not isolate patients with hepatitis C.^8^^,^ 12 ^,^ 22 Jadoul et al. demonstrated in one study the possibility of completely eradicating the C virus transmission within dialysis units through the adoption of universal precautionary measures, after reaching a 54-month follow-up with zero incidence of new cases of seroconversion contamination.^8^^,^ 32 ^,^ 33

Currently, nosocomial transmission is the main source of the C virus transmission, and several studies are devoted to finding the reasons for transmission in dialysis units.^34^ There are several hypotheses and, although it is not possible to exclude those related to the dialysis equipment and reuse, most of the data points to failures in following infection control protocols, such as preparation of medication at a contaminated site, reuse of medication in multiple patients, inadequate surface disinfection and failure to exchange gloves between patients. Inadequate hand washing, shorter shift time, and reduced numbers of technicians per patient also increase the risk of transmission.^22^^,^ 35 In addition, studies have shown that outbreaks of seroconversion occurred in patients who shared the same environment and not necessarily the same hemodialysis machine, emphasizing the possibility of transmission by healthcare professionals.^28^

**Figure 4:**
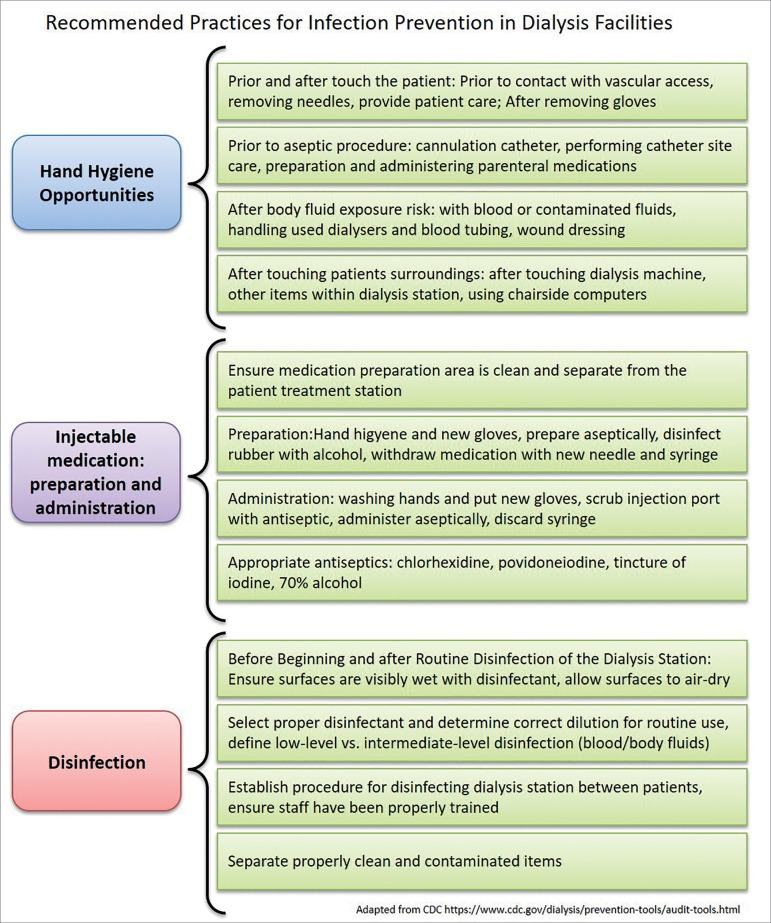
Universal precaution measures for safety in dialysis Source: Centers for Disease Control and Prevention. Control, C.f.D. and prevention, dialysis safety: audit tools, protocols and checklists, 2013.

The higher the number of years the patient has been in hemodialysis, the greater the risk of acquiring an HCV infection, taking into account the multiple exposures to the treatment during the week. It is important to stress that even if there is no visible blood on the surface, the HCV virus can remain potentially infectious on the surface for at least 16 hours. Studies analyzing the presence of non-visible blood and HCV-RNA on surfaces show high indices in several devices, such as hemodialysis machines, connectors, patient trays and fistula lavage sinks.^28^ In an epidemiological study with more than 4,000 patients from different dialysis units in the United States, they demonstrated a direct correlation between the incidence of hepatitis C and patient care by the healthcare team. After analysis, the main factors suspected as triggers of this episode were: inadequate cleaning of capillary boxes between uses, preparation of medications or stock of materials in areas where material contaminated with blood was handled and transport of injectable medications in mobile carts between patients.^33^^,^ 36 The CDC publishes on its website several checklists of infection control practices, all of which are important in reducing HCV transmission (Figure 4).^37^

The low compliance to universal precautionary measures is a constant in HUs around the world. A multicenter Spanish study with 9 Dialysis Units showed that in 93% of the opportunities, gloves are used, but only 36% of the staff sanitizes the hands after contact with the patient and 14% before contact. No differences in compliance to hygiene measures were observed among "white room" and isolation room staff.^38^ Similar data can be found in other observational studies from different localities and cultures.^39^^,^ 40

## Isolation

The isolation of patients with HCV infection emerged as an extension of the measures adopted for the isolation of patients infected with HBV, which, considering the characteristics of the two diseases, has no clinical basis. The 2008 KDIGO recommendations no longer advocate isolation of patients suspected of HCV infection. Strict compliance to HU infection control measures is best indicated as a preventive measure for contamination of other patients.^19^^,^ 41 Other protocols, such as the United Kingdom Renal Association and European Renal Best Practice, do not recommend isolation as a preventive measure.^42^^,^ 43

A recent Cochrane systematic review^44^ showed that the quality of evidence for or against isolation is very poor. Of the 123 papers evaluated, only one Randomized Controlled Trial (RCT) was found,^45^ and even in that study the level of evidence was considered low. In the single RCT carried out by Shamshiraz et al., which included 593 patients, there was no difference in the reduction of the HCV infection incidence with the use of exclusive machines; however, there are details in their methodology not described in this study that limit its use as a conclusive source stating the isolation ineffectiveness. Other publications adopt different isolation strategies that could be adopted and could be used in combination or separately, such as the exclusive use of machines, rooms, staff and shifts. Some of them show benefits from patient isolation; however, they are all observational studies and with inadequate evidence quality. In most of them, the study design is based on the intervention of isolation compared to its own historical controls, which creates a bias about the real reason for reducing incidence if it is directly related to the strategy or to the indirect effect of increased surveillance.^19^^,^ 44

The strategy of optimizing and strengthening universal care seems to be quite effective in controlling HCV infection, so many specialists suggest it as a primary measure, and the isolation is necessary when these practices are flawed. Observational studies show that isolation did not protect against HCV infection, and in the latter CDC guidelines such an attitude is not recommended.^12^ Favorable arguments for not isolating include the fact that the measure is not protective of other infections and creates, with segregation, a falsely protected environment against parenteral transmission; (HBV + HCV +, HBV + HVC-, HBV-HCV +, HBV-HCV-), the separation of patients with HBV and HCV can create logistic problems in the HUs. the isolation may predispose to reinfection by a second C virus genotype; the incubation period of HCV is long, and therefore many patients in the immunological window could be deemed uninfected; Finally, the creation of separate rooms increases the costs of dialysis, a sector that is already under-funded and has been struggling financially in recent years.^19^

The use of isolated machines is also not effective because, according to several studies with phylogenetic analysis, the highest risk of HCV is in patients who dialyze close to the infected patient, not in the same machine, emphasizing the importance of hygiene care between shifts, since the mechanism of transmission by single pass hemodialysis equipment makes the possibility of transmitting the virus by internal pathways remote, since it is not possible to pass through the intact membrane of the dialyzer. Therefore, the absence or failure to disinfect the surface of machines, armchairs and other equipment commonly used among patients is frequently identified as factors in the analysis of HCV outbreaks in HUs.^7^^,^ 19 ^,^ 46

In Brazil, by determination of the National Agency of Health Surveillance (ANVISA), there is no indication of a dedicated machine or isolation for HCV seropositive patients in a chronic hemodialysis program, who may remain in the same environment as their seronegative counterparts. Since 2014, all material used in the treatment must be used only once and discarded; In addition, disinfection and cleaning of surfaces between shifts is recommended in the HUs, in addition to general precautionary measures.^47^

## Treatment

Until recently, treatment possibilities for both hemodialysis and renal transplant patients are limited; dialysis patients often have low tolerance to interferon (IFN) and ribavirin (RBV) regimens, mainly due to anemia, while in renal transplant patients the use of IFN has been associated with the possibility of graft rejection.^48^^,^ 49

With the new direct acting antiviral drugs (DAA) and the free IFN regimens, a new perspective has been opened for patients with chronic kidney disease, enabling the achievement of high rates of sustained virological response (SVR) with very few adverse events and fewer drug interactions.^50^ When considering the use of DAA in interferon-free regimens, the degree of renal dysfunction of the patient should first be assessed, since not all drugs have evidence of being safe to use in patients with advanced renal dysfunction and in hemodialysis.

The recommendations are based on the glomerular filtration rate (GFR), which can be measured or estimated. If the estimated glomerular filtration rate (GFR) is used, the suggestion is to use the Chronic Kidney Disease Epidemiology Collaboration (CKD-EPI) formula for the calculation.^19^ Patients with CKD in conservative treatment and with GFR greater than 30 mL/ min may be treated with any of the drugs that are incorporated into the hepatitis C treatment regimens in our country: sofosbuvir, simeprevir, daclatasvir, ledipasvir, ombitasvir/veruprevir/dasabuvir combination (3D) and the combination grazoprevir/elbasvir, at the usual doses recommended for each genotype, in the same way as patients without renal dysfunction.

However, patients with GFR of less than 30 mL/min are restricted to the use of sofosbuvir, a renal elimination drug. To date, there is a limitation on the use, as indicated in the package insert, when the GFR is below 30 mL/min,^51^ due to the buildup of a metabolite (GS-3310007), which toxicity potential has not yet been fully elucidated. New studies will evaluate its use in more severe renal dysfunctions, identifying the best dose to be used and the possible dose interval, so that up to now the use of sofosbuvir in patients with GFR <30 mL/min should be done with caution, especially in pre-dialytic patients. In cases where there is an option for the use of sofosbuvir, it seems more appropriate to use the drug at a full dose (400 mg/day), associated with another antiviral (simeprevir, daclatasvir or ledipasvir, according to the genotype), since half dose or full dose on alternate days may be insufficient for treatment. For patients with genotype 3, the option is sofosbuvir associated with daclatasvir, for 12 weeks for non-cirrhotic patients, and for 24 weeks for cirrhotic patients.

Fortunately, there are other fairly safe options for patients with genotype 1 on dialysis. There are studies showing the safety of using the 3D combination in stage 5 chronic kidney disease on dialysis.^52^ Treatment for 12 weeks achieved 90% SVR in 20 patients who received the regimen. The regimen was safe, especially in patients with genotype 1b, who did not require the use of ribavirin. In studies with fewer patients with genotype 1a, the drug was also highly efficient without the use of ribavirin.^53^

Another very safe, effective and well studied regimen in patients with dialytic CKD is the grazoprevir and elbasvir association for 12 weeks. In the study with this combination, 115/116 patients obtained SVR, showing that this is an excellent option for the treatment of patients with genotype 1.^54^ For patients with genotype 3, since there is no alternative scheme without the use of sofosbuvir, it is recommended to use sofosbuvir associated with daclatasvir, with careful patient follow-up, although there is already enough data from the literature showing the safety of using this medication.^55^^-^ 57

A new combination of pangenotype drugs is in the final phase of incorporation into the treatment protocol in our country (Glecaprevir/Pribentasvir) and will also provide safety to dialysis patients with high response rates.^54^^,^ 58

The treatment regimens for hemodialysis patients adopted in Brazil are depicted on Tables 1, 2 and 3. In patients on dialysis, RBV should be used with great caution, starting at a dose of 250 mg/week, progressively, and in most cases it should not exceed the dose of 3 tablets of 250 mg/week.

**Table 1:** Treatment of patients with type 1a genotype under dialysis

	Time in treatment	Time in treatment	Time in treatment
	No cirrhosis	With cirrhosis Child A	With cirrhosis Child B/C
Elbasvir+Grazoprevir	12 weeks	12 weeks	Regimen not indicated
Regimen 3D	12 weeks + RBV	24 weeks + RBV	Regimen not indicated
Glecaprevir+Paritaprevir	8 weeks	12 weeks	Regimen not indicated

RBV: ribavirin; 3D: ombitasvir/veruprevir/dasabuvir

**Table 2:** Treatment of patients with type 1b genotype under dialysis

	Time in treatment	Time in treatment	Time in treatment
	No cirrhosis	With cirrhosis Child A	With cirrhosis Child B/C
Elbasvir+Grazoprevir	12 weeks	12 weeks	Regimen not indicated
3D Regimen	12 weeks	12 weeks + RBV	Regimen not indicated
Glecaprevir+Paritaprevir	8 weeks	12 weeks	Regimen not indicated

RBV: ribavirin; 3D: ombitasvir/veruprevir/dasabuvir

**Table 3:** Treatment of patients with type 2 and 3 genotypes under dialysis

	Time in treatment	Time in treatment	Time in treatment
	No cirrhosis	With cirrhosis Child A	With cirrhosis Child B/C
Glecaprevir+Paritaprevir	8 weeks	12 weeks	Regimen not indicated

Whatever the treatment schedule, the curing criterion is HCV-RNA negativity documented 3 months after the end of treatment.^7^^,^ 15 ^,^ 55 Despite the lack of consistent data in the literature, it seems reasonable to recommend that all cured patients could be allocated under the same conditions as the seronegative for hepatitis C and do without the need for dialyzer disposal, although there is no clear policy on this approach. What can be recommended is that cured patients who continue dialyzing in the same shift as untreated patients should perform the NAT every six months to detect possible reinfection.^19^

## Conclusion

The hepatitis C virus is still a frequent problem faced by nephrologist physicians within the dialysis units in our country, who are faced day by day with the interpretation of serological tests and the institution of measures that will protect any patients at risk of HCV contamination. This paper presents the SBN and SBH stances regarding points that should be debated in our routine, such as the need to extend and make feasible the diagnosis of HCV, not only for serology interpretation, but also for viral RNA analysis, which should be made available in our healthcare network in the specific situations described herein. In addition, greater synergism is needed between nephrologists and hepatologists, so that we can make available new drugs for the treatment of HCV in patients with CKD, especially those on dialysis, leading to cure and radically modifying the clinical outcome with respect to the results of renal transplantation and the development of chronic liver disease.
